# Perceptions of the influence of computer-mediated communication on the health and well-being of early adolescents

**DOI:** 10.1080/17482631.2017.1335575

**Published:** 2017-06-28

**Authors:** Lindsay Favotto, Valerie Michaelson, Colleen Davison

**Affiliations:** ^a^ Department of Public Health Sciences, Queen’s University, Kingston, Ontario, Canada; ^b^ School of Religion, Queen’s University, Kingston, Ontario, Canada; ^c^ Department of Emergency Medicine, Kingston General Hospital Research Institute, Kingston, Ontario, Canada

**Keywords:** Adolescent health, computer-mediated communication, well-being, social health, screen time

## Abstract

Recent technological advances have provided many youth with daily, almost continuous cell-phone and Internet connectivity through portable devices. Young people’s experiences with computer-mediated communication (CMC) and their views about how this form of communication affects their health have not been fully explored in the scientific literature. A purposeful maximum variation sample of young people (aged 11–15 years) across Ontario was identified, using key informants for recruitment. The young people participated in seven focus groups (involving a total of 40 adolescents), and discussed various aspects of health including the health impacts of CMC. Inductive content analysis of the focus group transcripts revealed two overarching concepts: first, that the relationship between health and the potential impacts of CMC is multidimensional; and secondly, that there exists a duality of both positive and negative potential influences of CMC on health. Within this framework, four themes were identified involving CMC and: (1) physical activity, (2) negative mental and emotional disturbance, (3) mindfulness, and (4) relationships. With this knowledge, targeted strategies for healthy technology use that draw on the perspectives of young people can be developed, and can then be implemented by parents, teachers, and youth themselves.

## Introduction

Canadian adolescents have become more and more connected via the Internet over the past 10 years, first through the medium of the desktop computer, and now more commonly through the use of a compact device such as a cell phone (mobile phone) or tablet. Using these devices in daily, and sometimes continuous, communication with friends is common (Steeves, 2014). The use of computer-mediated communication (CMC) allows young people to connect with their friends or family members regardless of physical distance. In a recent study of Canadian youth, 99% had access to the Internet in some fashion, and by fourth grade approximately 50% had their own cell phone (Steeves, 2014). With this high level of possible connectivity, the potential impacts of CMC on health are important to investigate.

Health has been most commonly defined as “… a state of complete physical, mental, and social well-being …” (World Health Organization, 2014). It has been characterized through physical, mental, and social domains (Huber et al., 2011): physical health encompasses the physiological processes that maintain homeostasis; mental health refers to emotions and psychological functioning, including being mindful of our thoughts and emotions and our ability to cope with stress; and social well-being includes the ability to form connections and relationships with others (Huber et al., 2011). Even though CMC is used mainly as a tool for communication and social interactions, the use of CMC has been linked to behaviours that can affect various aspects of one’s overall health.

Conflicting findings about potential impacts of CMC on health outcomes exist among scholars. With regard to social health, some scholars identify CMC as an opportunity for self-discovery, in which one tries on different personas outside the physical world (Turkle, 1999). Others explain the vast number of opportunities to communicate and build social supports with others (Greenhow & Burton, 2011; Kraut et al., 2002) as allowing the creation of diverse social networks (Hlebec, Manfreda, & Vehovar, 2006). These speculations are challenged by suggestions that the use of CMC has reduced face-to-face contact, and influenced feelings of loneliness and perceptions of social support (Kraut et al., 1998; Moody, 2001; Prezza, Pacilli, & Dinelli, 2004; Van Den Eijnden, Meerkerk, Vermulst, Spijkerman, & Engels, 2008; Vergeer & Pelzer, 2009). Furthermore, issues exist around physical and mental health in terms of young people’s sedentary screen time that is associated with CMC engagement. These include causing anxiety-provoking pressure for youth to always be accessible online in order to perfect their online persona (Bond, 2010; Leatherdale, 2010), and being digitally connected while at the same time unconnected to those in their physical environment (Turkle, 2008, 2012). The aforementioned potential benefits and potential drawbacks of using CMC among youth influence diverse aspects of their overall health.

There is little scientific literature that examines how young people experience, perceive, or assess the impact of CMC on their health. Youth are likely to have a unique perspective on the emerging phenomenon of CMC, and it is important to understand their perspectives about the benefits and drawbacks of these activities (Herring, 2008). This study investigates how the use of CMC could impact the health of young people in the early adolescent period, between the ages of 11 and 15 years, which is a stage of life in which CMC is increasingly common. We gathered the opinions and experiences of CMC among Canadian young people and explored their thoughts on how CMC affects their health-related behaviour. In doing so, we hope to highlight the youth perspective of this issue, as well as to develop a framework outlining the potential influence that this exposure may have on the health of young people overall. We also hope to explore and inform concrete ways in which we can promote the healthy use of technology for overall health benefits among early adolescents.

## Methods

### Data source and sample

This study used transcript data from seven youth focus groups conducted in 2014, involving 40 adolescents. A purposeful, maximum variation sampling strategy was used. This refers to a type of purposeful sampling wherein participates are recruited in a way that will maximize variability across identified characteristics (for us, these were sex, age, location in the province, and number of years in Canada). Common patterns that emerge from a very diverse sample of people are recognized as interesting and valuable (Patton, 2002). Key informants, who were well-situated people in the community, were able to contact potential participants according to the purposeful sampling plan and disseminate the study information (including informed parental consent forms). The final focus groups involved an Ontario-based, geographically diverse sample of youth, 11–15 years of age, and further detail about the focus groups has been previously provided (Michaelson, Mckerron, & Davison, 2015). Participants were recruited based on the characteristics of age, sex, rural/urban residence, and length of time living in Canada. A purposeful sample selects participants based on a strong ability to contribute towards understanding the overall phenomenon (Mayan, 2009). Within each focus group, participants were of homogeneous demographic characteristics, as they had one or more of the sampling characteristics in common (e.g. Group 1, all female; Group 2, all boys; Group 7, all recent immigrants to Canada). This was done to aid in facilitating conversation and comfort among the participants, as they shared similarities. The maximum variation was achieved through heterogeneity between all focus groups to create comparisons between groups and represent an overall diverse sample, including young people not born in Canada. Participants were sampled from eastern Ontario (Hastings and Frontenac Counties), northern Ontario (Greater Sudbury), western Ontario (Bruce County), and the Greater Toronto Area in central Ontario.

### Data collection

This study was inspired by the grounded theory methodology put forth by Glaser and Strauss (1967), in that rather than applying a predetermined theoretical perspective throughout data collection, an area of interest was identified, allowing findings to emerge “from the ground up” (Charmaz, 2014, p. 125). While the researcher begins with individual cases or experiences, increasingly abstract conceptual categories are then developed to synthesize, explain, and understand the data, as well as to identify patterns within the data. As theories begin to emerge throughout initial coding and analyses, subsequent questions are modified and focused. Earlier data allow the researcher to collect more data around emerging themes and questions.

Data were generated through asking youth broad questions around their perceptions of health. Themes that emerged early in the study helped to shape questions asked by trained facilitators in all subsequent focus groups (Michaelson et al., 2015). All audio from the focus groups was recorded and transcribed verbatim for analysis. Focus groups were used for data collection as large amounts of information can be collected in a short period (Berg, 2009; Morgan, 1997), with themes emerging organically from the discussion (Berg, 2009). Using focus groups for adolescent research specifically allows for the “shared culture” of this demographic to be revealed as the interactions and comparisons made between the young participants who engage with one another are captured (Morgan, 1997; Raby, 2010).

To initiate discussion in the focus groups, photo elicitation techniques were used. Generic photographs depicting aspects of social, physical, mental, and spiritual health to which the youth could relate, as well as images depicting youth, their friends, and their family members using technological devices, were introduced. This was undertaken to stimulate discussion beyond the use of probing questions and because “images evoke deeper elements of human consciousness than do words” (Harper, 2002, p. 13). The inclusion of images allowed for added ease of discussion during the focus group as the conversation was rooted in an image that both researcher and participant recognized (Harper, 2002). Given that the same preselected photographs were used in all the focus groups, thus incorporating preconceived ideas about health into the discussion, this study was not grounded theory in its purest form. Rather, it was inspired by grounded theory and used many elements of this method.

A professional transcriber who had signed a confidentiality agreement transcribed the audio recordings. As the use of personal narrative increases the risk to confidentiality (Fiske, 2009), all personal identifiers such as sex, location, and names of family members, friends, pets, and schools were removed from the transcript to protect the identity of participants. All transcripts (audio and written) were kept on password-protected computers. Participants were each given an “identifier code”, which linked to their demographic information. Transcripts and audio recordings were all stored in a locked cabinet in a locked office at Queen’s University. The key to connect the “identifier codes” with demographic information was encrypted on a password-protected computer, and a hard copy of this key was kept in a locked cabinet in a locked office in a separate building from where the demographic forms were stored.

### Analysis strategy

Discussions of CMC within the transcript were abstracted with two to three lines of transcript before and after the CMC discussion to ensure that context was available for the text of interest. Extraction excluded reference to video games, surfing the Internet, and general media. This allowed for the focus to be on communication technology. Inductive content analysis was undertaken on the remaining CMC-related focus group transcript data as per a method outlined by Elo and Kyngäs (2007). This is a method of qualitative analysis used to obtain an overall objective description of the phenomenon under study through the process of identifying categories that emerge from the transcript (Elo & Kyngäs, 2007). An inductive content analysis refers to the fact that the categories emerge to describe overarching concepts (Elo & Kyngäs, 2007).

The first level of coding involved line-by-line open coding to describe the explicit content of the text. This process involved reading each focus group transcript and tagging the meaning or content of each line briefly in the margins (Elo & Kyngäs, 2007). This level of coding allowed us to gain an early understanding of the basic content of the data (Mayan, 2009). The second and third authors co-coded two transcripts independently, then all three authors compared and discussed individual coding to be explicit about personal biases, and to allow for triangulation and transparency of codes. After these discussions, a consolidated code list was created and code definitions were written to ensure rigorous and streamlined labelling across all remaining focus groups. The total first order code list consisted of 79 codes or tags.

Following first order coding, all codes were grouped into meaningful units called categories (Forman, Damschroder, & Content, 2015). Categories were based on commonality among codes or identified links between code to reduce the overall quantity of codes and to develop meaningful units (Elo & Kyngäs, 2007; Graneheim & Lundman, 2004; Hsieh & Shannon, 2005). Formation of categories is a form of data simplification and brings added understanding to the text to begin describing the overall phenomenon (Elo & Kyngäs, 2007). In this second round of coding, 10 categories were established. Once all of the categories had been identified, quotations that corresponded to each category were reviewed to ensure homogeneity of transcript content to the identified category (Mayan, 2009). After the 10 main categories within the data had been developed, axial coding, which involved generating higher order themes, was applied (Mayan, 2009). Themes that linked multiple categories together within the categories were then identified, which led a higher level of integration (Graneheim & Lundman, 2004; Mayan, 2009). This third stage of analysis resulted in the identification of four core themes. Following this, two overarching concepts that linked all higher order themes and categories together at the highest level of analysis were identified. Emergent themes were assessed for strength based on the depth, consistency, and frequency of occurrence in all focus groups.

### Academic rigour

To enhance the trustworthiness of the findings, strong rigour was achieved in various ways. First, credibility was considered when selecting diverse participants based on demographics such as age, sex, urban/rural, and how long a young person has lived in Canada. This provided richness to the data gathered on the topic of interest (Graneheim & Lundman, 2004). Secondly, approaching the data through line-by-line open coding allowed the researchers to remain close to the data. Finally, constant collaboration with principal investigators who were involved in focus group data collection was maintained to provide triangulation between the researchers and to verify analysis (Graneheim & Lundman, 2004). The credibility of this study was further established by the inclusion of code descriptions and frameworks as part of the audit trail (Graneheim & Lundman, 2004). Concurrent memos in the form of technical or academic notes were continuously created to document thoughts, first impressions of the text, and early connections that were made by the researchers. These memos prevented loss of ideas, provided explicit information about personal biases, maintained the audit trail, and enhanced reflexivity. They also served as a map of the early analytic process and supported the overall inferences that were drawn from the data (Forman et al., 2015).

### Ethical considerations

Before participation in the focus groups, written and verbal information was given to parents and youth participants. The written consent of parents and written assent of participants were collected before participation in the study. In addition, at the start of each focus group, all youth participants provided verbal informed assent. The Queen’s Health Sciences and Affiliated Teaching Hospitals Ethics Board approved the focus group protocol (EPID-447-13-6,011,166) and this specific study (EPID-520-15-6,016,097).

## Results

The youth participants are described in Table I. All participants (*n* = 40) were students attending public schools throughout Ontario and all spoke fluent English.

**Table I.** Demographic characteristics of the study participants.

Characteristic	*n*
Sex	
Male	13
Female	27
Age (years)	
12–13	22
14–15	18
Residence in Ontario	
Rural	9
Rural/urban	17
Urban	14
Length of time in Canada	
Recent immigrant	7
Canadian born	33
Total participants	40

**Figure 1.**
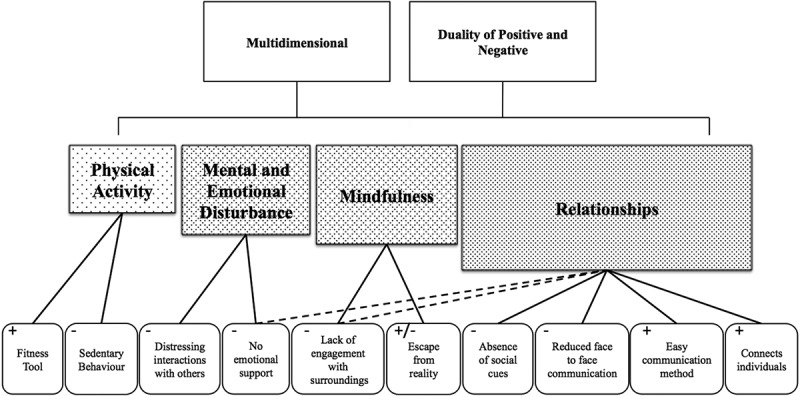
Categories, themes and overarching concepts of the relationship between computer-mediated communication (CMC) and the health of young people.

The content analysis of the CMC-specific sections of the transcript revealed a framework that included multiple themes around CMC use and the health of young people in Ontario (Figure 1).

Overall, this analysis identified two overarching concepts: (1) the multidimensional relationship between CMC and health, and (2) the duality of a positive and negative relationship between CMC use and health. This multidimensional relationship refers to the fact that engagement in CMC affects more than one domain of health. CMC can potentially influence social, mental, and emotional health, along with aspects of physical health. The second overarching concept of both positive and negative aspects suggests that the influence of CMC is two sided. There exists an element of duality here, as there are both benefits and drawbacks to use in regard to health. These two overarching concepts describe four themes: (1) physical activity, (2) emotional and mental disturbance, (3) mindfulness, and (4) relationships (Figure 1).

### Physical activity

#### Sedentary screen-time behaviour

Use of CMC was identified as having an impact on the time that youth remain sedentary, as it is a part of overall daily screen time. One youth expressed, “I think that is a big thing affecting health. Everyone now has a cell phone and computer and so people are less active …” (Group 6, female, urban). Another participant identified the use of cell phones as the opposite of a healthy and active lifestyle, stating that “they prevent us from going out and having [physical] activity” (Group 3, rural/urban).

#### Regulator of fitness

With smartphone technology and the advance of digital applications (apps) for health, the relationship between our devices and health is extended beyond communication. Individual ownership of cell phones and devices can aid us in regulating our fitness. As one participant told us:

Some phones or devices you can get a fitness set for it if you want. That could keep track of your weight or how much time you work out or what you have eaten and if it is healthy or not. (Group 6, female, urban)

This comment highlights the knowledge that this youth in particular recognized the capacity of these devices beyond a communication method and as a tool to monitor fitness trajectories.

### Emotional and mental disturbance

*Engaging in distressing interactions with others*. The use of CMC to connect with others evoked distressing negative emotions for some focus group members. One youth articulated that consistent engagement in CMC provided access to continuous negative and emotionally distressing interactions with peers:

I used to have it all of the time and I would [get] really angry and stuff. There was a lot of drama that was involved with it. People say stuff over text that they wouldn’t necessarily say in person. It made me more stressed and when I didn’t have it I felt better. (Group 1, female, rural/urban)

With the use of CMC, the ease of conversation resulted in hurtful interactions. Through constant connection through CMC, distance from her device was required to offer relief from these negative emotions.

Beyond the “drama” that was described as occurring between friends, many participants indicated that cyberbullying was a potential source of emotional distress in their lives. During the second focus group, one participant noted the influence of cyberbullying on youth, and described one of the pictures in the photo elicitation exercise like this: “she is looking at her phone sort of mad or sad maybe because of something like cyber bullying” (Group 2, male, rural). Furthermore, the lag time in messaging was a concern, as breaks in communication may leave questions unanswered and issues unresolved. One participant shared:

Text messages I find that I don’t know the person might feel okay but they are actually not. But when you text a message at 10:00 am and then again at noon hour that says yeah I am fine. And it is like well thanks for telling me hours later. I have been worrying. (Group 5, male, rural)

CMC allowed for connection at any point, but also lag time between connections when messages go unanswered. This break in communication resulted in some youth reporting distressing emotions. CMC can actively promote hurtful interactions among youth, along with passively affecting them as they wait for responses from their peers.

*No emotional support provided*. Negative communication not only caused mental distress for participants, but also was not always felt to be effective for providing support to others. This is illustrated by the following comment by a participant:

… if it is a personal conversation you are there to comfort them. If you are talking to someone on the phone or you are texting them and they start crying you can’t do anything. You are just there and the most you can tell them is that it is all right. That does not always work for people. (Group 7, female, urban)

This young person appeared to feel that she was unable to offer the proper support to a friend who was in need. Without being in the physical presence of one another, the innate features of text-based CMC provide a barrier in communication.

### Mindfulness

#### Lack of engagement with surroundings

When this sample of youth engaged in CMC, they often referred to experiencing a passive disengagement from the present moment. They reported a lack of mindful behaviour or lack of focus on other tasks after receiving important news through methods such as text messaging. One youth shared a bad experience by saying,

I got a phone call at my school saying that a family member passed away and I had to wait a couple of hours for my mom to get me and then I’d sit there and think … “I can’t focus on what is going on right now”. (Group 5, male, rural/urban)

Furthermore, there was an observed lack of awareness of the external environment when engaging in this method of communication. One participant stated that being completely engaged in CMC “is not healthy because they are not paying attention to what is around them” (Group 7, female, urban). Another participant articulated strongly that individuals who are fully engrossed in their technology are “hypnotized” (Group 7, female, urban) as when their own thoughts are so influenced by technology “you don’t really think or anything and that is all you know after a while” (Group 7, female, urban). These statements suggest that some youth have insight into the ability of communication technologies both to transport them from the present moment and to influence their ability to focus on present tasks.

*Escape from reality*. Another way this theme was discussed among the youth was in reference to opportunities for youth to actively “escape through electronics” (Group 3, rural/urban) and remove themselves from a situation or environment if they perceive it to be unhealthy or distressing. It can be their time to check out and not have to deal with negative situations of the present:

I feel that technology that we have can be a stress and at the same [time] it can bring you in to escaping what is going on around you and be an escape … Say if a kid is going through a tough time and it is not the greatest time and they are getting picked on at school. Or grandpa or grandma or mother or father is not doing so good … they escape what is going on around them and they are just into it and they are having a good time. (Group 3, rural/urban)

Disengaging from the present moment through CMC was a way of coping and bringing joy or a sense of calm to the youth when their environment was unhealthy or painful.

### Relationships

#### Easy communication method

Youth valued the influence of CMC in their lives. They articulated that it is “is easier to communicate” (Group 3, rural/urban) with CMC, as the innate features of CMC support quick and direct exchanges of communication between individuals.

#### Connects individuals

The use of CMC also provided benefits through allowing youth to “keep in contact with family who [do] not live close” (Group 4, rural), through allowing communication when physical distance would traditionally keep individuals distant. These modern media also enabled connection to others beyond the young person’s physical social network. When asked whether a technology medium such as YouTube was a barrier to real life, one youth responded:

Yes and no. You are really getting to know that person. When they get big on the YouTube they would [share] things about their personal life. They share positive and negative stuff. So it is kind of both. You can connect with them also. (Group 3, rural/urban)

CMC media allowed youth to connect to others who were physically distant or to those whom they knew solely from online platforms. This connection bridged social interactions from many points around the globe.

#### Absence of social cues

This theme related to the innate qualities of CMC that were lacking in comparison to face-to-face contact, and added another dimension to this study. One youth spoke of the fact that “by text message … you don’t know what their voice is like” (Group 5, male, rural/urban). This was a direct reference to the inability to hear auditory content in some CMC media such as text messaging and social media. Without these important social cues, “you don’t get to know what people are feeling and don’t see their emotions and all that stuff” (Group 7, female, urban). Furthermore, social cues were important for understanding the overall interaction:

When you are texting someone it is hard … when you talk to someone the tone of voice really tells what you are saying. You can say sorry genuinely or you can say, oh sorry. Over text it is hard to perceive what the person says. And that can cause drama because you can misunderstand things and there is miscommunication and stuff. It is good to have a balance. (Group 1, female, rural)

Without hearing tone of voice, a barrier was created in the effectiveness of the overall communication and support given to one another. With the potential for misunderstanding when engaging in CMC, this method of communication made conversation more difficult.

#### Reduced face-to-face communication

Not only do the features of these devices reduce closeness and interactions between people engaging in contact through CMC, but CMC was seen to reduce face-to-face social interactions with those around you: “It takes away from communication. You don’t communicate with people and you lose your ability to converse and … now-a-days most people can’t hold a proper conversation and it affects how you speak to people” (Group 7, female, urban). Some youth identified that face-to-face conversations have been negatively affected by the adoption of CMC.

A lack of conversing with people in the physical environment can potentially influence the relationships between individuals and their ability to connect. The use of devices in a way that hinders communication was identified as an obstacle to growing closer and building relationships. One participant noted that:

Electronics have become a big part of our lives and stuff. A lot of teenagers use them a lot. I am not saying that it is bad to go on electronics because it has helped a lot. But around a dinner Table 1 feel like you should be more with your family and talking to them. A lot of the time you are at school and your parents are at work and you won’t be able to see them until dinner. So that is a time to catch up with them. (Group 3, rural/urban)

Through this observation, this youth recognized that even though we live in a world surrounded by technology and CMC, there are certain times when it is important to connect with those around you face-to-face and not through CMC.

The importance of technology-free family time was a theme that emerged throughout all focus groups. One participant expressed, “If we are at the dinner table and we are on our phones, we are not talking about our day. So we don’t get closer as a family” (Group 4, rural). Another voiced that “There are times when you have to put it down and just talk to your family and not bring it to the dinner table” (Group 4, male, rural). Participants from all focus groups articulated that family time should be a time to converse together and build strong family relationships without the distraction of technology use by themselves or by their parents.

Overall, youth perceived that using CMC affected health behaviour in diverse ways. These findings were consistent across all focus groups, and were communicated by male and female participants and by youth living in urban and rural settings.

## Discussion

The aim of this study was to reflect on the perceptions of youth populations specifically, and to explore their ideas about the relationship between CMC and health. We observed that youth perceived that CMC affected behaviours that are linked to multiple domains of health. They also shared how those behaviours have the potential to affect health in both positive and negative ways.

### Physical activity

The young people in our study sample recognized the probable impact of CMC devices on their overall physical activity in both positive and negative manners. With regard to negative implications, youth expressed strong insight into the ability of CMC use to increase their sedentary behaviour. This finding accords with previous research. For instance, in Canada, it is recommended that youth spend less than 2 h per day sedentary, including time spent using CMC (Tremblay, Leblanc, Janssen et al., 2011). Among an Ontario-based sample of adolescents, 25% of these young people used social networking sites for more than 2 h per day, not including additional time spent using other methods of CMC (Koivusilta, Lintonen, & Rimpela, 2007). Sedentary behaviour is linked to poor physical health outcomes such as higher body mass index, along with low self-esteem and poor academic success (Tremblay, LeBlanc, Kho et al., 2011). The findings suggested that youth are aware of how CMC increases the time spent sedentary and how this is not beneficial for health. Even with this awareness, young people remain high users of CMC. Therefore, this knowledge does not appear to translate into a behavioural change for this population of users.

The second manner of probable influence of CMC use on physical activity, according to our participants, was in relation to positive impacts on behaviour. Youth acknowledged the potential of CMC devices to facilitate physical activity through fitness applications that can be downloaded to mobile devices. Adoption of this method to increase physical activity is suggested by Nicole, Evan, and Derikk (2015) for individual and school-based physical activity programmes, but evidence of the effectiveness of these applications on health remains mixed (Quelly, Norris, & Dipietro, 2015). With diversity in applications directed towards fitness, heterogeneous findings have emerged. Use has been found to increase enjoyment and motivation for fitness (Turner, Spruijt-Metz, Wen, & Hingle, 2015), but to produce insignificant changes to fitness and body composition (Quelly et al., 2015). Although participants suggested that these apps were beneficial, their clinical efficacy remains unclear. Given this, the influence of CMC devices on physical activity is not one-dimensional. Possible benefits include the ways in which fitness applications can connect individuals to active peer groups. However, the utility of these devices for physical fitness and health is unclear. Even though youth may see these devices as promoters of physical fitness in some situations, they may not be providing an overall benefit to health. Indeed, although not explicitly recognized by the youth in our study, the use of CMC for tracking fitness may lead to body image concerns and eating disorders. Overall, the discussion among the youth in this study regarding the impact of CMC on physical activity was predominantly towards sedentary influences. They were aware of a stronger pull of CMC to promote sedentary behaviours rather than physical activity. This may support the idea that some youth overestimate the potential of their devices to benefit physical fitness.

### Emotional and mental disturbance

Another category that emerged was the potential for negative emotional and mental health disturbances when engaging in CMC. The negative emotions such as stress, anger, and sadness that, according to the youth, resulted from engaging in CMC are also mentioned in previous research linking CMC with poor mental health outcomes. Use of CMC is associated with outcomes such as loneliness and low self-esteem (Chen & Lee, 2013; Sampasa-Kanyinga & Lewis, 2015) along with mental health issues such as depression and feelings of anxiousness (Riedl, Köbler, Goswami, & Krcmar, 2013; Sampasa-Kanyinga & Lewis, 2015). Cyberbullying was also mentioned as a concern as it elicits distressing emotions. Experiencing cyberbullying is consistently associated with rates of depression among young people (Hamm et al., 2015). This form of bullying can have traumatic influences on young people as the public platform allows for an unlimited number of viewers and constant engagement at any point throughout the day. The link between distressing emotions experienced by youth and cyberbullying has been described in previous research, emphasizing the strong mental and emotional health components of this problem. Youth are aware that the use of CMC has the potential to negatively impact their mental and emotional health. Even with this awareness, they still used this method of communication often to contact their peers. CMC has often become a social norm among groups of young people, even in early adolescence. The young people in our study indicated that negative emotional impacts did not lead to withdrawal from use. This finding points to the need for regulation of use to support strong emotional or mental health in young people.

In the focus groups, participants spoke of multiple situations at home, with their families, at school, or generally in their lives in which attention was taken from the present through someone’s use of technology. Mindfulness is being attentive to the present moment through being open, accepting, and curious about current experience (Hassed & Hassed, 2016). Innate traits of mindfulness (Curtiss & Klemanski, 2014), along with mindfulness-based training (Hofmann, Sawyer, Witt, & Oh, 2010), benefit mental health by improving situations of depression and anxiety among adults and youth (Kuyken et al., 2013). Our technology-rich society does not always support mindfulness or developing mindfulness skills among youth, and there exists a technology “co-presence” in which people are often removed from the present moment through technology (Turkle, 2008, p. 2). This allows for adoption of the “default mode”, which is characterized by feelings of distraction, lack of attention, and preoccupation in thoughts (Hassed & Hassed, 2016, p. 53), and is related to the ways in which technology promotes multitasking and divided attention between the virtual and physical world (Turkle, 2008). These ideas are well illustrated by the results of our study, which indicated that youth are removed from the present while engaging in CMC. Given that mindfulness is a skill that is beneficial to health (Hassed & Hassed, 2016), it is important for youth to limit distraction through CMC to support their overall mental health and well-being.

Youth also expressed distraction as a method of coping with poor current family or life situations. While many youth identified this emotion-focused repression coping style (Compas, 1987) as being beneficial, the literature does not point to an overall advantage of this coping style (Herman-Stabl, Stemmler, & Petersen, 1995; Mahmoud, Staten, Hall, & Lennie, 2012). Adolescents who cope using avoidant behaviours have significantly higher self-reported rates of depression (Herman-Stabl et al., 1995), anxiety, and stress (Mahmoud et al., 2012). Lack of awareness is linked to mental illnesses such as depression because it leaves the mind to ruminate on negative thoughts in a passive manner instead of drawing awareness to them (Ciesla, Reilly, Dickson, Emanuel, & Updegraff, 2012; Curtiss & Klemanski, 2014). Specifically among youth, the association between low mindfulness-based traits, or inability to focus on the present moment, and low mood was mediated by rumination (Ciesla et al., 2012). Therefore, youth may not acknowledge or even recognize the negative implications to their health in choosing to use CMC and technology as a distraction from real issues that they are experiencing. They see self-removal from the present negative situation as a solution for the short-term cessation of difficult emotions. Removal from the present situation through the use of technology may result in poor long-term influences and adoption of unhealthy coping behaviours early on in adolescence. In turn, this can influence coping and emotion-regulating abilities in the future.

### Relationships

Modern technology devices have allowed for communication between individuals regardless of physical distance (Monge & Contractor, 2003). Furthermore, these technologies have connected individuals who are separated by physical distance to the same virtual space, creating a sense of real gathering in a virtual world (Monge & Contractor, 2003). The youth in this study recognized and noted this benefit by referencing the ability of these technologies to bridge them to family and friends, even when they are apart. These findings are consistent with views that CMC benefits connections by allowing for an expansion of one’s social network and for often continuous interactions with those whom they do not encounter face-to-face (Hlebec et al., 2006; Lin, Sun, Lee, & Wu, 2007). This continuous connection can allow youth to feel closer to their friends (Valkenburg, Sumter, & Peter, 2011), and so benefit their social relationships and perceived social support. Young people value the ability to connect with friends, even when they are geographically apart, and communication with others regardless of physical distance has become a part of their social norm.

In contrast to the aforementioned benefits, CMC also poses barriers to social health. Youth consistently discussed the negative implications of CMC use on aspects of social health, indicating that this is probably the largest influence of CMC on overall health. Use of CMC was described as negatively influencing social health by affecting communication with others, and consequently influencing our relationships. Both communication and relationships have been altered through the adoption of CMC in society, and work hand in hand to impact our social networks and support from peers. These two aspects will be discussed further below.

Some participants acknowledged some innate and non-modifiable features of CMC that act as a barrier to communication. For example, the lack of social cues when using this method of communication affects the possible depth of connection. Previous research has identified that vocal cues are important for conversation, since without hearing a voice there is a high threat of perceived insincerity and conflict through misunderstanding someone’s true intention (Park, Chung, & Lee, 2012). The youth also noted that CMC reduced time spent engaging in face-to-face communication with others. In the past, it would be impolite to stop a face-to-face conversation with someone to check messages on your device or sit next to someone in a public space and not acknowledge the other person (Turkle, 2011, 2012). With individual technological devices, this has become accepted behaviour for many people (Turkle, 2011, 2012). This phenomenon can be explained through the displacement of social interaction hypothesis put forward by Kraut et al. (1998). This hypothesis outlines that through the integration of CMC into society, less communication is taking place face-to-face and, therefore, less satisfying relationships between individuals are being produced (Kraut et al., 1998; Turkle, 2011). With this displacement we have “confused connection with conversation” as small CMC-based interactions are not able to replace connection through conversation (Turkle, 2012). Warnings made by scholars regarding the reductions in conversation and poor communication quality associated with CMC were reflected in the perceptions of the youth in our focus groups. Even with these cautions by scholars, and the youth recognizing limitations as well, CMC continues to be used frequently for communication.

Participants also identified that the use of CMC influenced not only communication potential and relationship initiation between individuals, but also the quality of the relationships. Youth strongly referenced negative implications of CMC use while with their family members as it prevented them from engaging with one another and, in turn, they “don’t get closer as a family”. Research indicates that we do not have to be using our CMC device for it to influence relationships. The presence of a cell phone alone while individuals are interacting prevents meaningful conversation among individuals, reducing their perceived closeness to one another (Przybylski & Weinstein, 2012). In situations with strangers or even with family members, CMC provides a strong distraction to communication and building relationships. The youth in this study acknowledged communication as an important ingredient for strong relationships, especially with family. Strong family relationships can protect youth from engaging in risky behaviours such as alcohol use, smoking, and early sexual initiation, as well as providing support for youth experiencing mental health challenges (Viner et al., 2012). With concern over potential loss of connection due to CMC use, movement towards device-limited time with family members and friends may be one avenue for intervention that may be adopted by youth, and could support them in building strong relationships and social support.

Two overarching concepts identified through building on the perspectives of CMC use among youth are: (1) the multidimensional relationship between CMC and health, and (2) the duality of positive and negative potential influences for health. The way in which participants expressed the influences of CMC is consistent with a holistic view of health as it consists of multiple domains. This holistic view of health is reflected in the World Health Organization’s definition of health, as it identifies health as “… a state of complete physical, mental, and social well-being …” (World Health Organization, 2014). Participants expressed experiencing the potential influences of CMC in this holistic way, and this supports the continued adoption of these domains in regard to youth health promotion initiatives. In addition, the dual impact of both positive and negative elements is in line with the inconsistency shown in the literature. It showcases how there is no consensus in terms of CMC use being only harmful or only beneficial to health. The conflicting health beliefs around the influences of CMC present a challenge to future health promotion efforts targeting the behaviour change of withdrawing use, as there are not solely negative implications.

Even with recognition of harm on physical, mental, and social health, the use of CMC devices remains high among this cohort (Steeves, 2014). It is important to recognize that youth are aware of the potential detrimental health effects of CMC but also value the benefits. In conclusion, CMC can potentially influence health, but the active choices we make about the frequency and manner of use are the most important factors in determining the overall health influence. These choices can allow space for balance and control of the potential positive and potential influences on our health.

### Limitations

This study has specific limitations. The data were limited in scope since data collection was only conducted in Ontario, and these findings may not be transferable to all Canadian young people in this age bracket. Focus groups were conducted with grounded theory-inspired methodology (Michaelson et al., 2015), in the context of a study with a focus on holistic health. Therefore, CMC use by youth surfaced early on and was probed for in subsequent focus groups but was not an initial focus of data collection. This may reduce the depth of data regarding CMC, as it was not an initial aim of the focus groups. Limitations also exist within the focus group format itself. The group sessions were conducted at one point in time and therefore do not reflect changes in opinions or perspectives. With this method of data collection comes the potential for a few youth to dominant the discussion and data collection, preventing all opinions surfacing within the group, especially as focus group members were not strangers. This limitation was minimized through the involvement of trained facilitators who moderated all sessions. Potential biases involve the assumed directionality of CMC use potentially influencing health outcomes, along with an interest in the social implications of the relationship between CMC use and the health of youth.

### Strengths

The strengths of this qualitative study are the depth and richness of the data collected from the youth. The diverse male and female participants were recruited from a variety of ages and geographic areas throughout Ontario, and with different amounts of time spent living in Canada. All youth were engaged during the focus groups, eager to share their ideas and contribute to health science research. The richness of data also adds strength and trustworthiness to the findings. Trustworthiness was further supported by strong rigour, which was achieved through line-by-line coding, the development of an audit trail and triangulation between researchers. Triangulation involved independent coding of transcript sections by all authors to develop consistent code definitions and verify the analysis.

### Implications for interventions and further research

This study submits that multiple domains of health are related to CMC use in both positive and negative ways. There is a need to recognize the potential impact of CMC and technology use on health in a more holistic way. This study on CMC use and the health of young Canadians advances our awareness of possible health impacts that could be considered in future research to advance the discussion beyond youth safety while engaging with others online. Currently, there are limited resources for parents to support them in encouraging healthy and safe use of digital technology among their children. This study supports the adoption of health-promotion practices that recognize a wide range of aspects of health that are influenced by CMC. In addition, recognition of both the negative and the positive likely impacts of CMC on health will allow youth, parents, and teachers to recognize these influences and perhaps to identify ways to take steps in regulating CMC use in order to maximize the beneficial aspects for health and to control the negative ones. The American Academy of Pediatrics (2013) released a policy statement offering recommendations for parents regarding media use (American Academy of Pediatrics, 2013). They suggest limiting screen time to no more than 2 h per day through strategies such as having no Internet-connected devices in the bedroom along with developing a family plan for media use that establishes rules for the use of cell phones and Internet for all family members. These rules can include “curfews” for use around dinners and bedtime (American Academy of Pediatrics, 2013) and help to restore time for conversation, such as during car journeys or time spent with friends (Turkle, 2012). Participants in our focus groups recognized that the use of CMC should be limited in terms of both length of time and activities in which one is engaged. Having these discussions around setting limits to CMC use can contribute to the promotion of healthy CMC practices and best serve the overall physical, mental, and social health of Canadian adolescents.

## Conclusion

From the perspectives and opinions of the Ontario youth who participated in our study, the use of CMC has a diverse relationship with health. For these young people, engagement in CMC can potentially influence physical, mental, and social domains of overall health in both positive and negative ways. The discussion of negative implications of CMC for social health was most dominant. This method of communication connects people but negatively affects relationships through the absence of social cues necessary for effective and meaningful communication, along with reducing face-to-face engagement with others. The youth themselves strongly identified the need for face-to-face communication beyond CMC in order to grow closer to individuals. Based on these findings, we recognize the need for actively choosing to step away from our devices and encourage the reintroduction of face-to-face discussions in the schoolyards and in our homes, where they may have been lost. This action may reduce screen time and distraction, allow for the promotion of relationships with one another in the present moment, and aid in the building of social supports. Even though the dominant potential influence was negative, we recognize that there are beneficial aspects of CMC that need to be understood and enhanced to support the overall well-being of young people.
